# Evaluation modeling of highway collapse hazard based on rough set and support vector machine

**DOI:** 10.1038/s41598-022-23567-1

**Published:** 2022-11-04

**Authors:** Hujun He, Guorong Quan, Haolei Zhu, Wei Li, Rui Xing, Yichen Zhao

**Affiliations:** 1 School of Earth Science and Resources, Chang’an University, Xi’an, 710054 China; 2 Key Laboratory of Western Mineral Resources and Geological Engineering, Ministry of Education, Xi’an, 710054 China

**Keywords:** Environmental sciences, Natural hazards

## Abstract

The prediction of possibility and risk classification of collapse is an important issue in the process of highway construction in mountain area. Based on the principle of rough set and support vector machine, a landslide hazard prediction model was established. First of all, according to field investigation, an evaluation index system and a sample set of evaluation index data were established, the rough set decision table was constructed by preprocessing the original data based on the function classification of standard evaluation index, and then, the influence indexes of the collapse activity were reduced by rough set theory, and the main 9 indexes affecting the collapse activity as the key discriminant factors of support vector machine model, namely slope shape of slope, aspect of slope, slope of slope, height of slope, exposed structural face, stratum lithology, relationship between weakness face and free face, vegetation cover rate and weathering degree of rock were extracted. Then, taking the data of 13 post earthquake collapses in Yingxiu-Wolong highway of Hanchuan County measured by the authors in the field as training samples, the optimal model parameters were analyzed and calculated. When the penalty parameter $C$ is 8 and the kernel parameter $\sigma$ is 0.5, the correct rate of cross-validation is 100%, and the model is optimal. At last, 4 other landslide data were tested, the discriminant results of the test sample data were compared with the results obtained by uncertainty measure and distance discriminant analysis. The results show that the discriminant results of the test sample data by RS-SVM were consistent with the results obtained by uncertainty measure and distance discriminant analysis, the accurate rate is 100%. The collapse hazard analysis model based on rough set and support vector machine can reduce the computation while ensuring the accuracy of evaluation, and better solve the small sample and nonlinear problems, can provide certain a good idea for collapse hazard evaluation in the future.

## Introduction

Collapse is a geological phenomenon in which the rock and soil mass on a steep slope suddenly separates from the parent body under the action of gravity and other external forces to fall, roll and accumulates in a valley (or slope foot)^1–4^. With the deepening of the reform and opening-up and the implementation of the western development strategy, highway, railway and other projects continue to extend to the mountain area, high and steep slopes are more and more, so slope collapse and rockfall disasters are also increasing. In particular, the high and steep slopes after an earthquake, which are often unfavourable for earthquake resistance, are prone to collapse and rockfall disasters. For example, the 5.12 earthquake (2008 Wenchuan earthquake) triggered a large number of collapses, seriously damaging the transportation infrastructure such as highways etc., has greatly affected the disaster area people’s production and life and earthquake relief work. Therefore, it will provide strong support for regional geological disaster assessment and sustainable development of geological environment to carry out the evaluation and prediction of highway collapse geological disaster by information, quantification and science.

Because of the numerous and uncertainty factors that affect the collapse activity, many methods for predicting and evaluating the collapse geological hazards have emerged. For example, Liu^5^ applied the analytic hierarchy process (AHP) and the fuzzy comprehensive evaluation method to evaluate the risk of collapse disaster, Zhang et al.^6^ used the factor weighted summation model of the improved analytic hierarchy process to evaluate the sensitivity of landslides induced by the earthquake of Beichuan County, Xue et al.^7^ proposed the risk evaluation model of collapse disaster based on extension theory and fuzzy theory, Gao et al.^8^ constructed a landslide collapse risk assessment model based on GIS and information quantity model, He et al.^9,10^ established a comprehensive evaluation model of collapse hazard based on uncertainty measure and an information entropy and distance discriminant analysis model for predicting the grade of collapse hazard, Liu^11^ used Newmark displacement calculation model and probability method etc. to evaluated the risk of landslide induced by volcanic eruption of Changbai mountain pool in the sky. Broeckx et al.^12^, Greco and Sorriso-Valvo^13^, Mandal and Mondal^14^, Yang et al.^15^ used logistic regression model to evaluate and predict the susceptibility of landslides in the study area. Feng^16^ used the weight of evidence model, information model and logistic regression model to evaluate the susceptibility of landslides in Shilou-Jixian section of the middle and lower reaches of the Yellow River based on ArcGIS platform etc.

However, with the deepening of the research, it is found that the indexes affecting the activity of highway collapse are quite complex, which include the internal characteristics of the collapse body itself, such as elevation, slope direction, slope, stratum lithology, exposed structural face, soil type, etc., there are also external factors such as groundwater, precipitation, rock weathering, earthquake and various human activities that induce collapse disasters. Some of these indexes are redundant and have nothing to do with the evaluation results. When the above-mentioned mathematical theory method was used for evaluation, the attribute importance degree of several indexes was not analyzed in order to optimize the evaluation indexes, especially, as a non-linear, multi-level, fuzzy and complex system, and the conditions of highway collapse are different in different areas, it is difficult to obtain the complete collapse index data. As a scientific research of intelligent computing, the rough set (RS) theory can be used to analyze the attribute dependence degree of many indexes that affect the highway collapse, delete the relatively unimportant attributes, and achieve the goal of index optimization, and the weight distribution of the reduced indexes is carried out. As a pattern recognition method of minimize structural risk based on statistical theory, the support vector machine (SVM) can deal with the objective and practical problems such as small sample or finite sample, non-linearity and so on.

However, the SVM can’t determine which knowledge in the data is useful and which is redundant when processing the training samples, so the dimension of the information space can’t be simplified. Thus, when the dimension of input information space is large, the training needs a long time, which will reduce the real-time performance of the prediction system. The RS theory can find the relationship between the data without any prior knowledge. It can’t only remove the redundant input information, but also simplify the spatial dimension of input information. However, the RS theory is sensitive to noise when dealing with practical problems, so the result of training samples without noise is not good in noisy environment. The SVM has a better ability to suppress noise^17–22^.

Therefore, in this paper, the RS theory and SVM technology are combined to construct a mathematical model for comprehensive assessment and prediction of highway collapse risk. Based on the data analysis of the collapse of Yingxiu-Wolong highway in Hanchuan County of Sichuan Province after the 5.12 earthquake in Wenchuan County, the spatial dimension of the input information, which is the initial index, is reduced and optimized, find out the key index system that affects the evaluation and forecast of highway collapse risk, and remove the irrelevant index. On this basis, more purposeful and targeted research on this section of the highway has been carried out again to obtain the survey data, and apply the support vector machine model to carry out a comprehensive evaluation of the risk of collapse in this section of the highway. The good results are obtained, it is significant to evaluate and forecast the risk of highway collapse.

## Rough set theory

Rough set theory^23–27^ as a scientific study of intelligent computation was put forward by the Polish mathematician Pawlak in 1982, which is a set of mathematical theory methods for expression, learning, induction, etc. of incomplete data, imprecise knowledge. The essence of this theory is attribute reduction. It is well known that when the data in the knowledge expression system (information system) is collected at random, there is general redundancy. Decision table is a knowledge expression system with conditional attribute and decision attribute. A knowledge representation system can be expressed as $S = \left( {U,A,V,f} \right)$, where, $U$ is a finite set of objects, $A = C \cup D$ is a finite set of attributes, $C$ and $D$ is a set of conditional attributes and a set of decision attributes, respectively, $V$ is a domain of attributes $A$, $f:U \times A \to V$ is an information function, refers to the property value of each object. In the knowledge expression system, the importance degree of attributes is different. In condition of keeping the classification ability of knowledge base unchanged, according to the relevance in the set of conditional attributes, the goal of attribute reduction is to find some important condition attributes, which make the classification of decision attributes consistent and uniform.

Reduction is usually not unique, and all or minimum reduction in finding attributes has been proved to be an NP (non-deterministic polynomial)-hard problem. At present, the rough set attribute reduction algorithms mainly include Johnson greedy algorithm, exhaustive algorithm, attribute importance heuristic algorithm, genetic algorithm, dynamic reduction, concept lattice and so on. The exhaustion algorithm is to simplify the resolution function derived from the resolution matrix by the absorption law, and make it a minimum disjunctive normal form to obtain the reduction of the data attribute set. Considering that the exhaustion algorithm is only suitable for small data sets, and all reduction can be obtained despite the complexity of the algorithm, so the paper adopts the exhaustion algorithm to simplify the evaluation index of highway collapse risk grade.

## Support vector machine

In 1995, Corinna Cortes and Vapnik first proposed the support vector machine (SVM)^19,26,28–37^, in which supervised learning models for classification and regression analysis are related to related learning algorithms, they can analyze data and identify patterns. SVM allows for the optimal classification of linear and non-linear separable data.

**Figure 1:**
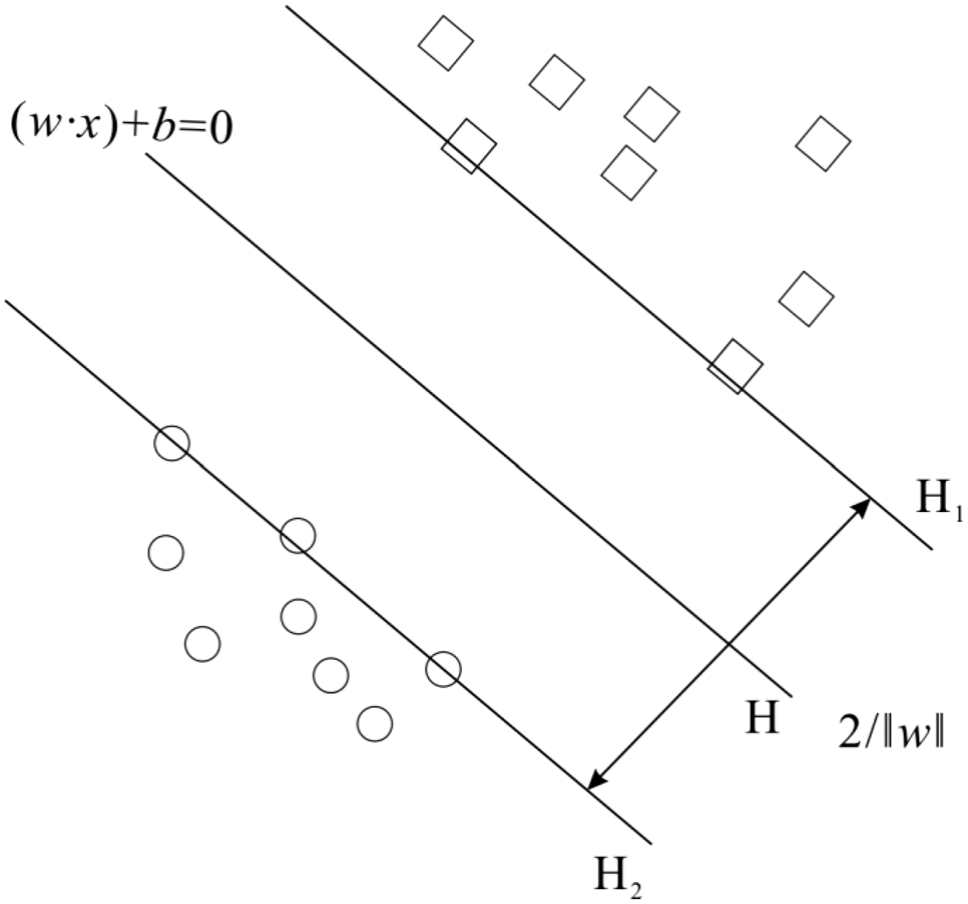
Schematic diagram of the optimal classification surface of two-type linear classification.

The basic idea of SVM classification is to map $x_{i}$ in a given set of samples $T = \left\{ {\left( {x_{1} ,y_{1} } \right),\left( {x_{2} ,y_{2} } \right), \ldots ,(x_{l} ,y_{l} )} \right\}$, $x \in R^{n} ,y \in \left\{ { - 1,1} \right\}$ to a high-dimensional feature space (Hilbert space) by a nonlinear mapping $\phi \left( \cdot \right)$, in this feature space, the maximum optimal classification hyperplane $w \cdot \phi \left( x \right) + b = 0$ for the classification interval $2/\left\| w \right\|$ is constructed to separate exactly the two kinds of points in the training sample set (Fig. 1). The standard SVM determines $w$ and $b$ by solving Eq. (1):

1 $$ \begin{aligned} & \min \frac{1}{2}\left\| w \right\|^{2} { + }C\sum\limits_{i = 1}^{l} {\xi_{i} } \hfill \\ & s.t.\;\;y_{i} [w \cdot \phi (x_{i} ) + b] \ge 1 - \xi_{i} ,\xi_{i} \ge 0,\;i = 1,2, \ldots ,l \hfill \\ \end{aligned} $$

where $C$ is the penalty parameter,$\xi_{i}$ is the relaxation variable.

The dual form of Eq. (1) is

2 $$ \begin{aligned} & \max \sum\limits_{i = 1}^{l} {\alpha_{i} } - \frac{1}{2}\sum\limits_{i = 1}^{l} {\sum\limits_{j = 1}^{l} {\alpha_{i} \alpha_{j} y_{i} y_{j} K\left( {x_{i} ,x_{j} } \right)} } \hfill \\ & s.t.\;\;\sum\limits_{i = 1}^{l} {\alpha_{i} y_{i} } = 0,C \ge \alpha_{i} \ge 0,i = 1,2, \ldots ,l \hfill \\ \end{aligned} $$

where $K\left( {x_{i} ,x_{j} } \right) = \Phi \left( {x_{i} } \right) \cdot \Phi \left( {x_{j} } \right)$ is the kernel function. In order to realize the linear classification of nonlinear variation, the kernel functions commonly used are Gauss radial basis function (Rbf), polynomial kernel function (Poly), Fourier kernel function (Fourier), multilayer neural network kernel function (Sigmoid) and so on.. The optimal solution $\alpha^{*} { = }\left( {\alpha_{1}^{*} , \ldots ,\alpha_{l}^{*} } \right)^{{}}$ is obtained by solving Eq. (2), and then $b^{*} = y_{j} - \sum\limits_{i = 1}^{l} {\alpha_{i}^{*} } y_{i} K(x_{i} ,x_{j} ),0 < a_{i}^{*} < C$ is calculate to obtain the classification decision function is

3 $$ f\left( x \right) = {\text{sgn}} \left\{ {\sum\limits_{i = 1}^{l} {\alpha_{i}^{*} } y_{i} K\left( {x_{i} ,x_{j} } \right) + b^{*} } \right\} $$

## Rough set-support vector machine model for risk evaluation of highway collapse

**Figure 2:**
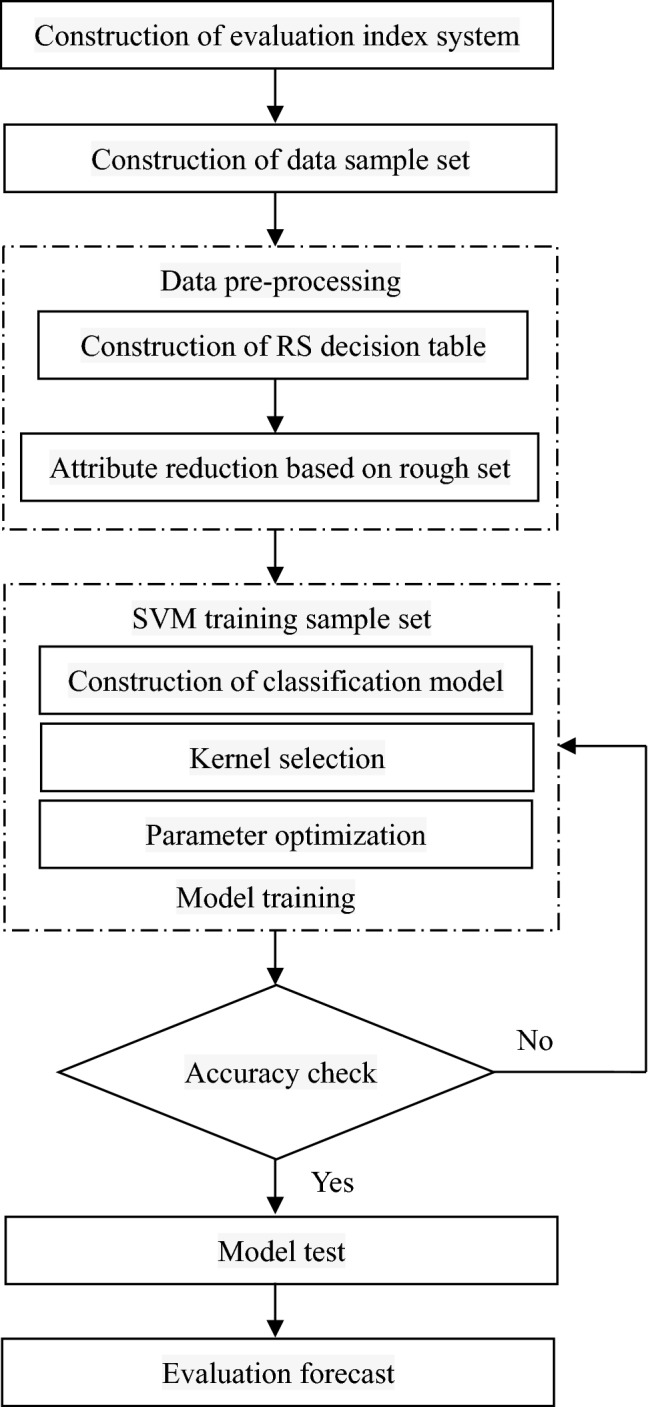
Flow diagram of highway landslide hazard evaluation based on RS-SVM.

Firstly, the factors affecting the risk of highway collapse are comprehensively analyzed, the key evaluation indexes are selected, the evaluation index system is constructed. The data sample set of evaluation indexes is constructed by data collection and field investigation. According to the grading standards of the evaluation indexes, the RS decision table is constructed by preprocessing the original data, and the RS attribute reduction is used to eliminate the redundant and unimportant attributes, so as to extract the features of highway collapse risk information. Then, training samples and test samples are selected from the data preprocessed by reduction, and the suitable kernel functions are selected and parameters are optimized. Support vector classification is trained with training samples, RS-SVM highway collapse risk assessment model is established to evaluate the risk level of the test evaluation samples. The specific flow of highway collapse risk assessment based on RS-SVM is shown in Fig. 2.

### Index system of highway collapse risk assessment

**Figure 3:**
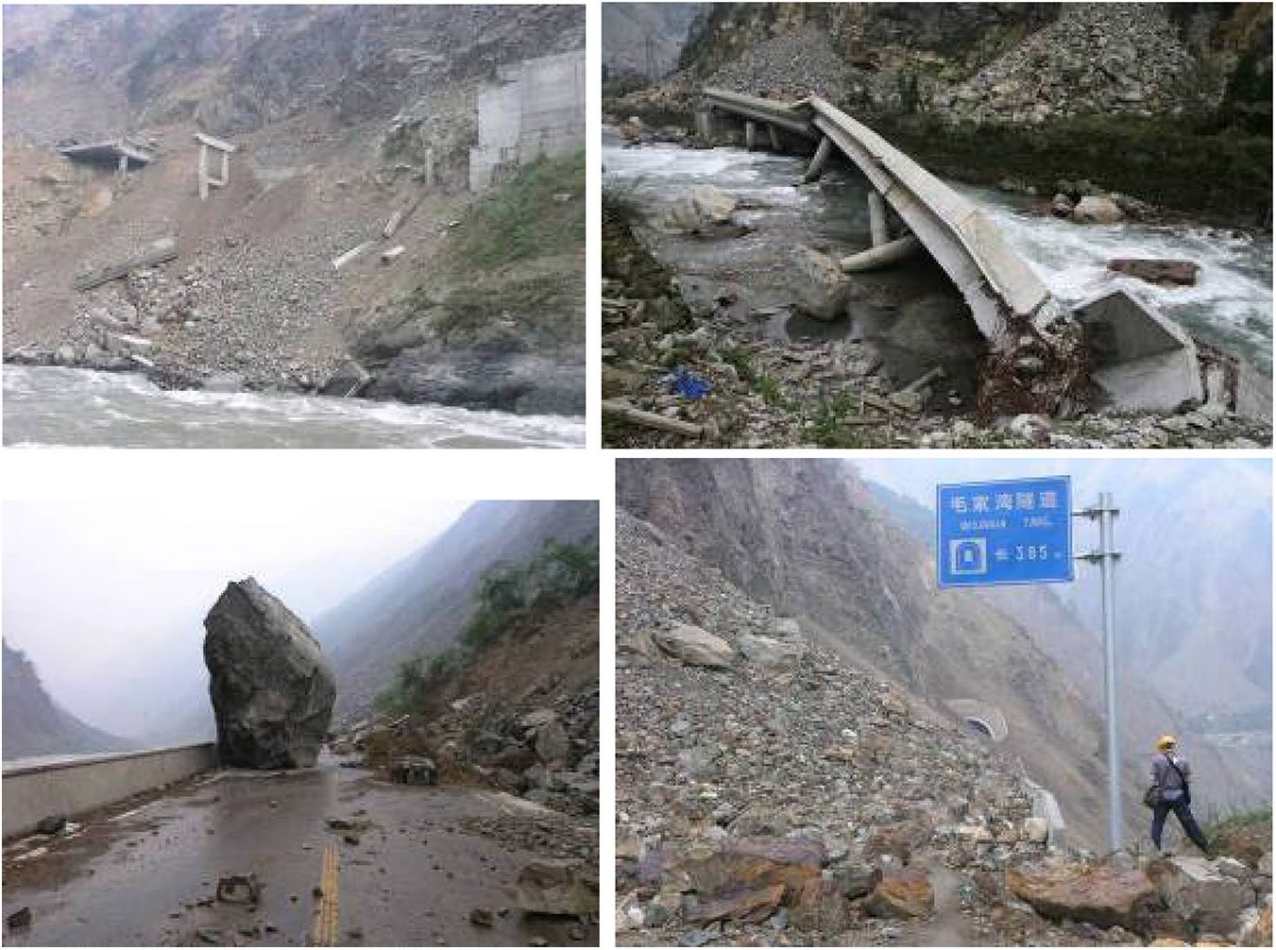
Slope rock mass landslide in 5.12 Wenchuan earthquake destroys a bridge, blocks the road, buries smashes tunnel entrance.

The S303 Yingxiu-Wolong highway in Hanchuan County, Sichuan Province was paved before the May 12, 2008 earthquake. The highway’s total length is 45.5 km and it is an important trunk route connecting Yingxiu and Wolong, As the closest highway to the epicenter of the Wenchuan earthquake, the highway is basically routed along the Longmenshan tectonic belt, starts from the Beichuan-Yingxiu fault (the central fault) and passes through the Maoxian-Hanchuan fault (the Houshan fault) in Longmenshan. It has complicated geological conditions, is the earthquake geological disaster most development, the damage is most serious a highway (Fig. 3). In order to comprehensively and objectively analyze and evaluate the geological disaster of highway collapse, the acquisition of information knowledge of highway collapse disaster is the key to complete the collapse risk assessment. The aim is to select the most effective knowledge from the original field geological survey data, eliminate redundant information, reduce the dimension of feature space, and improve the generalization ability of the evaluation system. Therefore, when constructing the index system, in order to achieve the establishment completeness and comprehensiveness of the comprehensive evaluation index system, we must first ensure that the index system has a broad generalization. Therefore, according to the field investigation data of highway collapse, on the basis of comprehensive analysis and study on the evaluation indexes of highway collapse risk at home and abroad, the paper combines with the key factors affecting the risk of highway collapse to determine 4 grades and 15 index items finally (Table 1). The evaluation index system of highway collapse risk is constructed using 15 indexes such as $x_{1}$,$x_{2}$,$x_{3}$,…,$x_{15}$.

**Table 1:** Evaluation factors and grading standard of highway landslide hazard.

Evaluation index		Evaluation grades			
		I (*C*_*1*_)	II (*C*_*2*_)	III (*C*_*3*_)	IV (*C*_*4*_)
Topography	(*x*_*1*_) Slope shape of slope	Fold line slope (4)	Concave slope (3)	Straight slope (2)	Convex slope (1)
	(*x*_*2*_) Aspect of slope	Sunny slope (4–3)		Shady slope (2–1)	
	(*x*_*3*_) Slope of slope/°	> 60	40–60	20–40	< 20
	(*x*_*4*_) Height of slope/m	> 150	100–150	50–100	< 50
Geologic structure	(*x*_*5*_) Meso structure	Development (4)	More development (3)	Less development (2)	No Development (1)
	(*x*_*6*_) Exposed structural face	Development (4)	More development (3)	Less development (2)	No Development (1)
Stratum	(*x*_*7*_) Stratum lithology	Loose rock (4)	Cemented chasten (3)	Well cemented half hard (2)	Hard rock (1)
	(*x*_*8*_) Weakness interlayer	Clear (4)	More clear (3)	Less clear (2)	No clear (1)
	(*x*_*9*_) Relationship between weakness face and free face	Consequent slope (4)	Oblique crossing (3)	Cross slope (2)	Reverse slope (1)
Climatic and hydrological geological condition	(*x*_*10*_) Mean annual rainfall/ mm/year	> 1500	1000–1500	500–1000	< 500
	(*x*_*11*_) Rainfall erosion/m	> 0.5	0.3–0.5	0.1–0.3	< 0.1
Other factors	(*x*_*12*_) Vegetation cover rate/%	< 5	5–15	15–30	> 30
	(*x*_*13*_) Weathering degree of rock/%	> 30	10–30	5–10	< 5
	(*x*_*14*_) Earthquake intensity/°	> 8	5–8	3–5	< 3
	(*x*_*15*_) Human activity intensity	Big (4)	Bigger (3)	Lesser (2)	Small (1)

In the whole evaluation index system, semi-quantitative method and measured value are used to evaluate the qualitative and quantitative indexes respectively. The classification standards and descriptions are shown in Table 1. The result of comprehensive evaluation is divided into 4 grades, which are expressed by I, II, III, IV respectively. Considering that there are many evaluation indexes in the comprehensive evaluation index system, and the contribution of each evaluation index is different in the whole evaluation, in order to reduce the decentralization of weight and the calculation workload, the redundant influencing factors should be removed firstly. In the paper, RS theory is used to reduce the whole index system, extract the key influencing factors, and then evaluate the index system based on SVM.

### Attribute reduction of risk evaluation index of highway collapse

The paper selects 17 collapse points from S303 Yingxiu-Wolong highway as the research object, and obtains the related original index data of collapse points through collecting and field investigation^9,10^. Based on RS theory, first of all, the original data should be discretized by the grading standard of evaluation index (Table 1), in which each index is divided into 4 grades according to the evaluation index system, we can get the rationality two-dimensional information evaluation decision table of the comprehensive evaluation system (Table 2).

**Table 2:** Discrete evaluation data tables.

Evaluation index		Collapse data																
		BT05	BT09	BT13	BT24	BT33	BT40	BT49	BT54	BT58	BT66	BT70	BT80	YBT03	W06	W17	W28	W33
Topography	(*x*_*1*_) Slope shape of slope	1	3	3	2	1	1	2	3	2	4	1	2	3	4	4	3	2
	(*x*_*2*_) Aspect of slope	3	1	3	3	1	3	1	3	3	1	3	1	3	3	1	1	3
	(*x*_*3*_) Slope of slope/°	4	3	4	2	3	4	4	3	2	3	4	3	3	3	3	2	4
	(*x*_*4*_) Height of slope/m	2	4	3	4	3	1	4	1	4	4	2	4	2	1	3	4	1
Geologic structure	(*x*_*5*_) Meso structure	1	1	1	1	1	1	1	1	1	1	1	1	1	1	1	1	1
	(*x*_*6*_) Exposed structural face	2	2	3	2	2	1	3	1	2	1	2	2	2	1	1	2	1
Stratum	(*x*_*7*_) Stratum lithology	3	3	2	3	2	2	3	4	2	2	3	3	4	4	3	3	3
	(*x*_*8*_) Weakness interlayer	1	1	1	1	1	1	1	1	1	1	1	1	1	1	1	1	1
	(*x*_*9*_) Relationship between weakness face and free face	4	2	3	3	1	2	3	2	4	3	2	3	3	4	3	3	4
Climatic and hydrological geological condition	(*x*_*10*_) Mean annual rainfall/ mm/year	3	3	3	3	3	3	3	3	3	3	3	3	3	3	3	3	3
	(*x*_*11*_) Rainfall erosion/m	2	2	2	2	2	2	2	2	2	2	2	2	2	2	2	2	2
Other factors	(*x*_*12*_) Vegetation cover rate/%	3	2	2	2	2	1	3	2	2	2	2	2	3	2	3	3	3
	(*x*_*13*_) Weathering degree of rock/%	2	2	2	2	2	2	2	3	3	2	4	3	3	3	3	3	3
	(*x*_*14*_) Earthquake intensity/°	4	4	4	4	4	4	4	4	4	4	4	4	4	4	4	4	4
	(*x*_*15*_) Human activity intensity	2	2	2	2	2	2	2	2	2	2	2	2	2	2	2	2	2
Decision attribute value		2	3	2	3	3	3	2	3	3	3	3	3	2	3	2	2	2

Then, based on the Rosetta data analysis software developed by the scientists and technicians of Warsaw University in Poland and Norwegian University of Science and Technology, the attribute reduction of the decision table is carried out by the exhaustive algorithm, after removing six very small redundant indexes such as $x_{5}$, $x_{8}$, $x_{{{10}}}$, $x_{11}$, $x_{14}$ and $x_{15}$, nine key indexes such as $x_{1}$, $x_{2}$, $x_{3}$, $x_{4}$, $x_{6}$, $x_{7}$, $x_{9}$, $x_{12}$ and $x_{13}$ are obtained.

### Sample set of evaluation model based on rough set and support vector machine

Using the data of 17 collapse points on both sides of S303 Yingxiu-Wolong highway, which is reduced by rough set theory, a sample set based on the support vector machine model is constructed, the first 13 samples are selected as training samples from 17 samples, and the best RS-SVM model is constructed. The other 4 samples (collapse number is W06, W17, W28 and W33) are used as test samples (Table 3).

**Table 3:** Evaluation value of evaluation indexes.

Collapse number	Evaluation index									Risk grade
	$x_{1}$	$x_{2}$	$x_{3}$	$x_{4}$	$x_{6}$	$x_{7}$	$x_{9}$	$x_{12}$	$x_{13}$	
BT05	1	3	70	73	3	3	4	6	7	II
BT09	3	1	50	613	2	3	2	18	8	III
BT13	3	3	69	105	4	2	3	20	6	II
BT24	2	3	30	234	2	3	3	25	6	III
BT33	1	1	48	119	2	2	1	25	6	III
BT40	1	3	85	21	1	2	2	35	8	III
BT49	2	1	70	189	4	3	3	8	9	II
BT54	3	3	42	25	0	4	2	20	12	III
BT58	2	3	28	314	3	2	4	25	15	III
BT66	4	1	47	410	1	2	3	20	8	III
BT70	1	3	68	66	2	3	2	25	40	III
BT80	2	1	45	200	2	3	3	20	12	III
YBT03	3	3	42	83	3	4	3	8	15	II
W06	4	3	43	30	0	4	4	25	15	III
W17	4	1	50	162	1	3	3	12	25	II
W28	3	1	40	214	2	3	3	6	28	II
W33	2	3	72	28	1	3	4	6	25	II

In this paper, the SVM model is implemented by the latest open source libSVM package in Python environment, which is developed by Chih-Chung Chang and Chih-Jen Lin^38^. The form of kernel function and the determination of its parameters determine the performance and complexity of the classifier, but there is no effective way to choose the best kernel function for a specific problem and to determine the parameters of kernel function. Considering that the number of support vectors in the Gauss radial basis function is less and the number of iterations is the least, we uses Gauss radial basis function $K\left( {x_{i} ,x_{j} } \right) = exp\left( { - \frac{{\left| {x_{i} - x_{j} } \right|^{2} }}{{2\sigma^{2} }}} \right)$ for the penalty parameter $C$ and kernel function parameter $\sigma$ in the SVM model, the grid search method is used to get the best parameters of combinatorial optimization. In the paper, the simulation is done in Python environment, and the penalty parameter $C$ = 8 and the kernel parameter $\sigma$ = 0.5 are determined by testing the training samples, the correct rate is 100%, and the model is optimal.

In order to verify the correctness and reliability of highway collapse risk discrimination based on the RS-SVM model, the RS-SVM model was used to distinguish training samples BT05 to YBT03. The results are shown in Table 4. All the 13 samples are correctly identified with a misjudgement rate of 0. The other 4 samples are tested according to the RS-SVM model studied well. The results are shown in Table 5. The results obtained by the uncertainty measures and distance discriminant methods are listed in Table 5. The results based on RS-SVM model are in good agreement with those obtained by many other methods, and are completely in line with the actual situation, with an accuracy rate of 100%. In order to further verify the superiority of RS-SVM, we input the same raw data without RS pre-processing into SVM for training and testing. The comparison of prediction result between SVM and RS-SVM are shown in Table 6. The results show that the multi-classification prediction model based on RS-SVM does not need too much prior knowledge and learning samples, but also too many parameters to be adjusted in the prediction model, can bring convenience to training and study. At the same time, under the same computer configuration, the training time of SVM model with RS pre-processing is significantly shorter than that of RS-SVM model, and the accuracy of the test is higher. Therefore, the application of RS-SVM classification model in the highway collapse risk prediction and evaluation is feasible, with high classification effectiveness and accuracy, which can provide useful reference for the practical projects.

**Table 4:** Training samples of RS-SVM model.

Collapse number	Evaluation index									Risk grade			
	$x_{1}$	$x_{2}$	$x_{3}$	$x_{4}$	$x_{6}$	$x_{7}$	$x_{9}$	$x_{12}$	$x_{13}$	Actual grade	The method of this paper	Uncertainty measure	Distance discriminant method
BT05	1	3	70	73	3	3	4	6	7	II	II	II	II
BT09	3	1	50	613	2	3	2	18	8	III	III	III	III
BT13	3	3	69	105	4	2	3	20	6	II	II	II	II
BT24	2	3	30	234	2	3	3	25	6	III	III	III	III
BT33	1	1	48	119	2	2	1	25	6	III	III	III	III
BT40	1	3	85	21	1	2	2	35	8	III	III	III	III
BT49	2	1	70	189	4	3	3	8	9	III	III	III	III
BT54	3	3	42	25	0	4	2	20	12	III	III	III	III
BT58	2	3	28	314	3	2	4	25	15	III	III	III	III
BT66	4	1	47	410	1	2	3	20	8	III	III	III	III
BT70	1	3	68	66	2	3	2	25	40	III	III	III	III
BT80	2	1	45	200	2	3	3	20	12	III	III	III	III
YBT03	3	3	42	83	3	4	3	8	15	II	II	II	II

**Table 5:** Test samples of RS-SVM model.

Collapse number	Evaluation index									Risk grade			
	$x_{1}$	$x_{2}$	$x_{3}$	$x_{4}$	$x_{6}$	$x_{7}$	$x_{9}$	$x_{12}$	$x_{13}$	Actual grade	The method of this paper	Uncertainty measure	Distance discriminant method
W06	4	3	43	30	0	4	4	25	15	III	III	III	III
W17	4	1	50	162	1	3	3	12	25	II	II	II	II
W28	3	1	40	214	2	3	3	6	28	II	II	II	II
W33	2	3	72	28	1	3	4	6	25	II	II	II	II

**Table 6:** Comparison of prediction result between SVM and RS-SVM.

Prediction algorithm	Training time (s)	Testing time (s)	Classification accuracy (%)
SVM	0.037082	0.034955	88.24
RS-SVM	0.034878	0.032544	100

## Conclusions

1. In the evaluation and prediction of highway collapse risk, firstly, rough set theory is used to reduce the indexes, and the relatively unimportant indexes are deleted, so as to achieve the goal of index optimization. Then, the 9 key indexes which affect the collapse activity, such as slope shape of slope, aspect of slope, slope of slope, height of slope, exposed structural face, stratum lithology, relationship between weakness face and free face, vegetation cover rate and weathering degree of rock, are extracted to be used as the discriminant factors of the support vector machine classification model.
2. The actual survey data of 17 collapse points optimized from Yingxiu to Wolong section of the S303 highway are selected as the training and testing samples of the support vector machine, the best parameters of combinatorial optimization are obtained through the training of the training samples, the RS-SVM model is used to evaluate and predict the test samples.
3. The research results show that the highway collapse discriminant analysis model based on RS-SVM achieves the goal of index optimization, not only can guarantee the accuracy of evaluation, but also reduce the computational load of the model, the learning performance is good, and the prediction accuracy is high, it is an effective method to forecast and evaluate the risk of highway collapse.

## Data Availability

All data generated or analyzed during this study are included in the paper.
